# Prognostic significance of BMI after PCI treatment in ST-elevation myocardial infarction: a cohort study from the Swedish Coronary Angiography and Angioplasty Registry

**DOI:** 10.1136/openhrt-2020-001479

**Published:** 2021-02-15

**Authors:** Shabbar Jamaly, Bjorn Redfors, Elmir Omerovic, Lena Carlsson, Kristjan Karason

**Affiliations:** 1 Department of Cardiology, Sahlgrenska University Hospital, Gothenburg, Sweden; 2 Department of Molecular and Clinical Medicine, Institute of Medicine, Sahlgrenska Academy at University of Gothenburg, Gothenburg, Sweden; 3 Transplant Institute, Sahlgrenska University Hospital, Gothenburg, Sweden

**Keywords:** obesity, coronary artery disease, percutaneous coronary intervention

## Abstract

**Background:**

Obesity along with clustering of cardiovascular risk factors is a promoter for coronary artery disease. On the other hand, a high body mass index (BMI) appears to exert a protective effect with respect to outcomes after a coronary artery event, termed the obesity paradox.

**Methods:**

The Swedish Coronary Angiography and Angioplasty Registry collects information on all patients who undergo percutaneous coronary intervention (PCI) for ST-elevation myocardial infarction (STEMI) in Sweden along with demographic and procedure-related data. We studied the predictability of four categories of BMI for 1-year all-cause mortality in people with STEMI undergoing PCI.

**Results:**

Among 25 384 patients, mean (SD) age 67.7 (12.1) years and 70.2% male, who underwent PCI for STEMI, a total of 5529 (21.8%) died within 1 year. Using normal weight (BMI 18.5–24.9 kg/m^2^) as a reference, subjects with obesity (BMI ≥30 kg/m^2^) had a low 1-year all-cause mortality risk in unadjusted analysis, HR 0.59 (95% CI 0.53 to 0.67). However, after adjustment for age, sex and other covariates, the difference became non-significant, HR 0.88 (95% CI 0.75 to 1.02). Patients with overweight (BMI 25.0–29.9 kg/m^2^) had the lowest 1-year mortality risk in analysis adjusted for age, sex and other covariates, HR 0.87 (95% CI 0.79 to 0.97), whereas those with underweight (BMI <18.5 kg/m^2^) had the highest mortality in both unadjusted HR 2.22 (95% CI 1.69 to 2.92) and adjusted analysis, HR 1.62 (95% CI 1.18 to 2.23).

**Conclusion:**

The protective effect of obesity with respect to 1-year mortality after coronary intervention became non-significant after adjusting for age, sex and relevant covariates. Instead, overweight people displayed the lowest risk and underweight individuals the highest risk for adjusted all-cause mortality.

**Trial registration number:**

NCT02311231.

**Key questions**

**What is already known about this subject?**

- Obesity along with clustering of cardiovascular risk factors is a promoter for coronary artery disease. On the other hand, a high body mass index (BMI) (>30 kg/m^2^) appears to exert a protective effect with respect to outcomes after a coronary artery event, termed the obesity paradox.

**What does this study add?**

- In unadjusted analysis, subjects with a BMI higher than 30 kg/m^2^ had the lowest 1-year all-cause mortality after percutaneous coronary intervention (PCI) for ST-elevation myocardial infarction when compared with people with lower BMI categories. However, after adjusting for age, sex and covariates, the protective effect of obesity became non-significant. Instead, people with overweight (BMI 25–29.9 kg/m^2^) showed the lowest risk for 1-year all-cause mortality.

**How might this impact on clinical practice?**

- We conclude that the apparent protective effect of obesity post-PCI could be due to confounders and speculate that a greater muscle mass together with enhanced cardiorespiratory fitness are more important with respect to outcomes after a coronary event.

## Introduction

Obesity, together with associated clustering of cardiovascular risk factors, such as hypertension^1^ dyslipidaemia^2^ and diabetes,^3^ is a strong promoter for cardiovascular disease morbidity and mortality.^4–6^ Weight control is considered to be of fundamental importance in primary prevention aimed at reducing the overall incidence of cardiovascular disease^7^ and is also targeted in secondary preventive programmes intended to improve outcome in patients with established cardiovascular disease.^8 9^

Still, a certain hesitancy has arisen concerning the beneficial effects of weight loss as a secondary prevention practice since several epidemiological studies have suggested that obesity may be protective in patients with coronary artery disease undergoing intervention.^10–12^ The apparent favourable effect of obesity on outcomes after coronary interventions, known as the ‘obesity paradox’, has generated a substantial amount of controversy.^13^ A protective effect of excess body fat is somewhat counterintuitive and a mechanism involving reverse causality has been suggested.^14^ Also, body fat distribution, cardiorespiratory fitness and unintentional weight loss could constitute confounders accounting for the somewhat paradoxical relationship between obesity and prognosis after coronary intervention.^15^

The aim of the present study was to evaluate the relationship between body fatness, divided up as four different body mass index (BMI) categories, and mortality in a large Swedish population undergoing percutaneous coronary intervention (PCI) due to an ST-elevation myocardial infarction (STEMI).

## Patients and methods

### Patient population

The Swedish Coronary Angiography and Angioplasty Registry (SCAAR) was established in 1992 and contains information about all coronary angiographies and PCIs (formerly known as angioplasty with stent).^10^ Each catheterisation procedure is described with approximately 50 angiography and 200 PCI variables, including both demographic and procedure-related data. The registry is financed by the Swedish government and the Association of Local Authorities and Regions and is supported by the Swedish Heart Association, the National board of Health and Welfare and the Swedish Heart and Lung Foundation

All consecutive patients undergoing PCI for STEMI in Sweden between 1 January 2011 and 31 May 2018 were included in the study. STEMI was defined according to the European Society of Cardiology criteria^16^ as a condition when there is evidence of myocardial injury defined as a dynamic change in cardiac troponin values with at least one value above the 99th percentile upper reference limit, or a persistent chest discomfort suggestive of myocardial ischaemia, along with an ST-segment elevation in at least two contiguous leads. Body weight and height, either measured or self-reported, were entered in the register at the time of the intervention. BMI, as a measure of nutritional status, was calculated as the weight in kilograms divided by the square of the height in metres. Other patient characteristics and information on comorbidities were imported into the register from medical records. All patients admitted to the cardiac care unit are informed both verbally and in writing about their participation in the registry. The investigation conformed with the principles outlined in the Declaration of Helsinki.

### Outcome measures

The primary endpoints were mortality rates at 30 days and at 1 year. All Swedish citizens have a specific personal identity number that is recorded in connection with all healthcare contacts and makes it feasible to follow how the Swedish population interacts with the healthcare system. The SCAAR registry obtains data on patients’ vital status from the Swedish Cause of Death Register, which originates from 1952 and includes the cause of mortality for all of citizens registered in Sweden at the time of their death.^17^

### Statistics

Statistical analyses were performed with SAS V.9.4 statistical software packages (SAS Institute, Cary, NC). Study participants were divided into four categories according to their nutritional status as recommended by WHO^18^: underweight (BMI <18.5 kg/m^2^), normal weight (BMI 18.5–24.9 kg/m^2^), overweight (BMI 25.0–29.9 kg/m^2^) and obese (BMI ≥30.0 kg/m^2^). Data are presented for the total study population and for each BMI group separately as means and SDs, medians and IQRs, or numbers and percentages. Comparisons between groups at baseline based on a complete case analysis were performed with ANOVA for normally distributed numerical data, χ^2^ test for categorical data and Kruskal-Wallis rank-sum test for non-normally distributed data. The number of complete cases with data for each variable is given as the denominators in table 1.

**Table 1:** Baseline characteristics for the total study group and for the different BMI categories separately*

Characteristics	Total group (n=25 384)	Underweight (n=244)	Normal weight (n=8488)	Overweight (n=11 132)	Obese (n=5520)	P value
Age (years)	67.7±12.1	74.8±11.6	71.1±12.0	67.1±11.5	63.6±11.7	<0.001
Weight (kg)	81.2±16.0	48.2±5.9	68.4±9.2	82.7±9.5	99.7±14.5	<0.001
Height (cm)	172.9±9.7	167.0±8.9	172.2±9.3	173.9±8.9	172.3±10.6	<0.001
Body mass index (kg/m^2^)	27.2±6.1	17.3±1.1	23.0±1.6	27.3±1.4	33.8±9.4	<0.001
Female sex	28.9% (7345/25 384)	68.9% (168/244)	33.8% (2870/8488)	23.4% (2606/11 132)	30.8% (1701/5520)	<0.001
Hypertension	49.0% (12 221/24 952)	43.8% (103/235)	43.9% (3648/8309)	48.6% (5330/10 973)	57.8% (3140/5435)	<0.001
Hyperlipidaemia	25.5% (6325/24 792)	19.0% (44/231)	21.5% (1780/8266)	25.8% (2810/10 899)	31.3% (1691/5396)	<0.001
Diabetes mellitus	16.2% (4095/25 218)	7.5% (18/241)	11.3% (955/8427)	15.0% (1666/11 072)	26.6% (1456/5478)	<0.001
Insulin-treated diabetes mellitus	7.0% (1756/25 169)	3.7% (9/241)	4.7% (396/8413)	6.0% (666/11 057)	12.6% (685/5458)	<0.001
S-creatinine (μmol/L)	92.4±55.1	89.2±65.8	92.4±55.7	91.3±50.9	92.0±53.0	<0.001
eGFR <30 mL/min/ m^2^	1445±5.7	24±9.8	558±6.6	576±5.2	287±5.2	<0.001
Previous renal failure	2.5% (640/25 384)	2.9% (7/244)	2.7% (227/8488)	2.1% (237/11 132)	3.1% (169/5520)	0.003
Previous dialysis	0.4% (107/25 384)	0.4% (1/244)	0.4% (37/8488)	0.4% (43/11 132)	0.5% (26/5520)	0.875
Current smoker	28.6% (6880/24 033)	41.4% (94/227)	29.9% (2388/7990)	27.0% (2857/10 577)	29.4% (1541/5239)	<0.001
Previous COPD	5.3% (1355/25 384)	21.3% (52/244)	6.4% (546/8488)	4.1% (456/11 132)	5.5% (301/5520)	<0.001
Previous cancer diagnosis	2.3% (590/25 384)	3.3% (8/244)	2.6% (222/8488)	2.2% (250/11 132)	2.0% (110/5520)	0.073
Previous dementia	0.4% (96/25 384)	1.6% (4/244)	0.4% (38/8488)	0.4% (43/11 132)	0.2% (11/5520)	<0.001
Previous heart failure	4.4% (1120/25 384)	6.6% (16/244)	4.7% (395/8488)	3.7% (414/11 132)	5.3% (295/5520)	<0.001
Previous myocardial infarction	13.0% (3296/25 384)	11.9% (29/244)	12.6% (1073/8488)	12.6% (1402/11 132)	14.3% (792/5520)	0.017
Previous PCI	10.8% (2750/25 384)	7.4% (18/244)	9.7% (827/8488)	11.0% (1220/11 132)	12.4% (685/5520)	<0.001
Previous CABG	2.9% (747/25 384)	1.2% (3/244)	2.5% (214/8488)	3.0% (334/11 132)	3.6% (196/5520)	0.002
Previous stroke	31.9% (7661/24 033)	22.5% (51/227)	27.9% (2229/7990)	33.8% (3579/10 577)	34.4% (1802/5239)	<0.001
Prior peripheral vascular disease	3.4% (864/25 384)	11.9% (29/244)	4.1% (347/8488)	3.0% (335/11 132)	2.8% (153/5520)	<0.001
No of days hospitalised	4.0 (3.0, 5.0)	4.0 (3.0, 8.0)	4.0 (3.0, 6.0)	4.0 (3.0, 5.0)	4.0 (3.0, 5.0)	<0.001
Acute occlusion	60.9% (15 460/25 384)	54.1% (132/244)	60.4% (5129/8488)	61.4% (6830/11 132)	61.0% (3369/5520)	0.464
Intraprocedural thrombosis	19.7% (4988/25 376)	16.4% (40/244)	19.0% (1609/8487)	20.5% (2278/11 128)	19.2% (1061/5517)	0.052
Successful procedure	95.3% (24 017/25 194)	91.5% (215/235)	95.2% (8016/8420)	95.8% (10 595/11 061)	94.8% (5191/5478)	0.847
Aspirin pre-cath lab	95.3% (24 017/25 194)	91.5% (215/235)	95.2% (8016/8420)	95.8% (10 595/11 061)	94.8% (5191/5478)	0.782
Clopidogrel pre-cath lab	19.0% (4814/25 366)	18.9% (46/244)	19.3% (1638/8484)	19.4% (2153/11 126)	17.7% (977/5512)	0.118
Ticagrelor pre-cath lab	58.1% (13 800/23 736)	54.6% (125/229)	56.9% (4515/7941)	58.5% (6077/10 385)	59.5% (3083/5181)	0.198
Prasugrel pre-cath lab	4.1% (1032/25 368)	2.9% (7/244)	4.0% (342/8484)	4.2% (465/11 127)	4.0% (218/5513)	0.705
Warfarin pre-cath lab	2.1% (526/25 368)	2.0% (5/244)	2.2% (185/8484)	2.0% (223/11 127)	2.0% (113/5513)	0.863

Data are presented as mean±SD, percentages (numerator/denominator) or medians (IQRs).

BMI, body mass index; CABG, coronary artery bypass grafting; COPD, chronic obstructive pulmonary disease; eGFR, estimated glomerular filtration rate; PCI, percutaneous coronary intervention.

For the first primary endpoint, participants were followed until death or for 30 days; and for the secondary primary end point, participants were followed until death or up to 1 year. At these time points, the SCAAR and Cause of Death Register were linked. Death is presented as a cumulative incidence function and comparison between groups was performed with the log-rank test. Persons who emigrated or were alive at the end of the 1-month or 12-month follow-up, respectively, were treated as censored observations. Death within the first 30 days was included as an event in the analysis for outcome at 1-year follow-up since we felt that that the risk for bias was low and, therefore, a landmark analysis redundant. Furthermore, since there were no competing events, it was not necessary to take this into account in the statistical analysis.

To evaluate the association between BMI categories and mortality, univariable and multivariable-adjusted HRs were calculated using Cox proportional-hazards regression models. We performed a primary investigation employing a complete case analysis. Thereafter, to handle missing data, we performed a secondary examination after multiple imputation missing pattern (MIMP) with the missing at random (MAR) assumption. The first model was unadjusted, the second model was adjusted for age, the third model was adjusted for age and sex, and the fourth and final model was adjusted for all covariates listed in table 1. The reference group was defined as the normal nutritional category as defined by WHO corresponding to a BMI of 18.5–24.9 kg/m^2^. All models were specified prior to conducting analyses and adjusted for preselected baseline risk factors considered of importance for the outcome. Further, the causal directed acyclic graph approach was applied when adjusting for confounding. An adjusted mortality analysis for the first 30 days was also considered, but due to few events, we concluded that the study was underpowered for this. Penalised spline regression was applied to study relationship between BMI as a continuous variable and all-cause mortality. The likelihood ratio test was used to examine the consistency of the association between BMI categories and mortality in the following subgroups defined by baseline characteristics: males versus females; age >65 years versus age ≤65 years; presence or absence of diabetes; smokers versus non-smokers. All statistical tests were two-tailed and p values of <0.05 were considered statistically significant.

## Results

### Patient characteristics

Between 1 January 2011 and 31 May 2018, a total of 25 384 patients underwent coronary artery catheterisation for STEMI at 29 PCI centres in Sweden. Among these, a total of 1304 (5.1%) died within 30 days of PCI and 5529 (21.8%) died within 1 year after the intervention. Baseline characteristics for the total study group, and for different BMI categories separately, are presented in table 1. People with obesity tended to be younger and have a more adverse cardiovascular risk factor profile with higher frequencies of hypertension, hyperlipidaemia and diabetes as compared with those in other BMI categories, but were less often smokers than those who were underweight. Underweight patients were more often females, smoked more frequently and had a higher prevalence of chronic obstructive pulmonary disease (COPD). The angiographic burden of coronary artery disease and number of days hospitalised were similar among patients with different BMI categories.

### Unadjusted 30-day and 1-year mortality in different BMI classes

**Figure 1:**
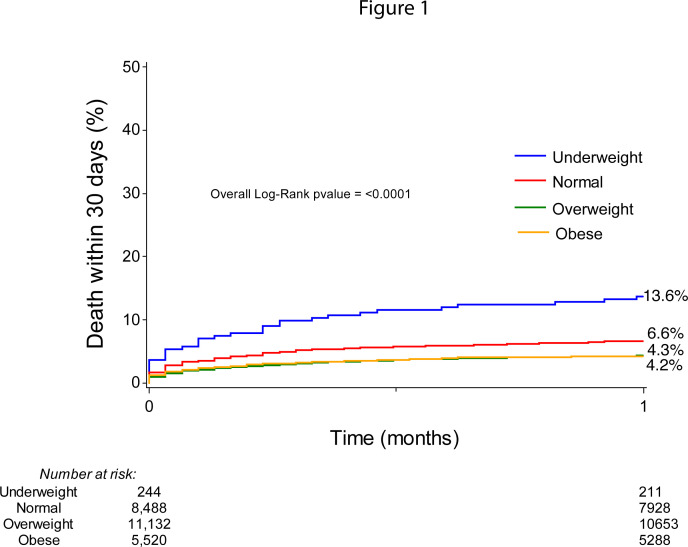
Thirty-day all-cause mortality after percutaneous coronary intervention in STEMI for different body mass index categories.

Unadjusted *30-day* all-cause mortality for different BMI categories are presented with cumulative incidence curves in figure 1. Patients who were underweight had the highest 30-day mortality (13.3%), followed by patients with normal weight (6.6%). Overweight and obese patients had somewhat lower cumulative mortality (4.3% and 4.2%, respectively). The overall log-rank p value was <0.001.

**Figure 2:**
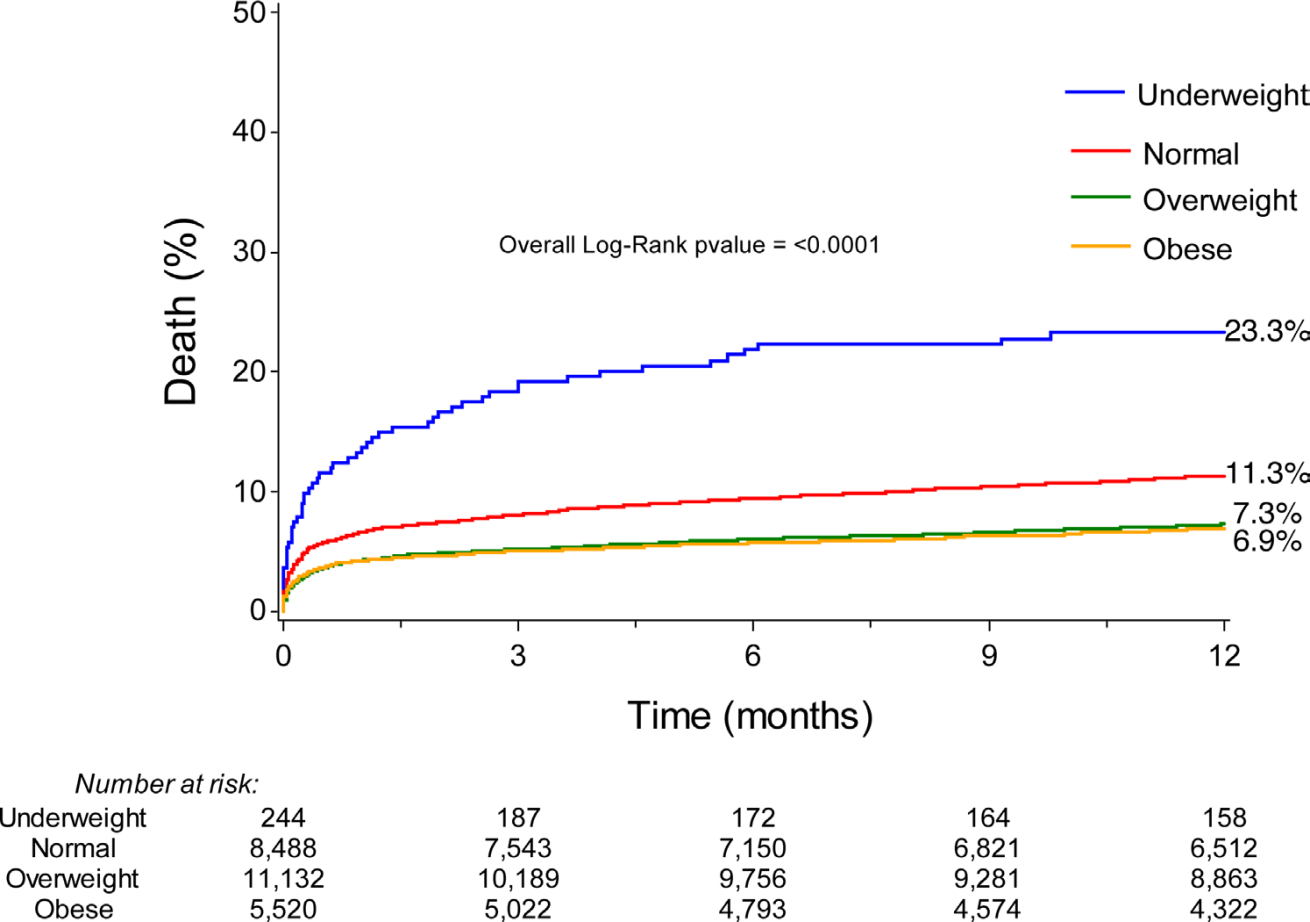
One-year all-cause mortality after percutaneous coronary intervention in STEMI for different body mass index categories.

Unadjusted *1-year* all-cause mortality for different BMI categories is presented with cumulative incidence curves in figure 2. Again, there was a substantial difference in mortality between different BMI classes with the highest risk in the underweight group (23.3%) followed by those with normal weight (11.3%). Patients who were overweight or obese had a lower 1-year mortality risk (7.3% and 6.9%, respectively). The overall log-rank p value was <0.001.

### Adjusted 1-year mortality in different BMI categories

**Figure 3:**
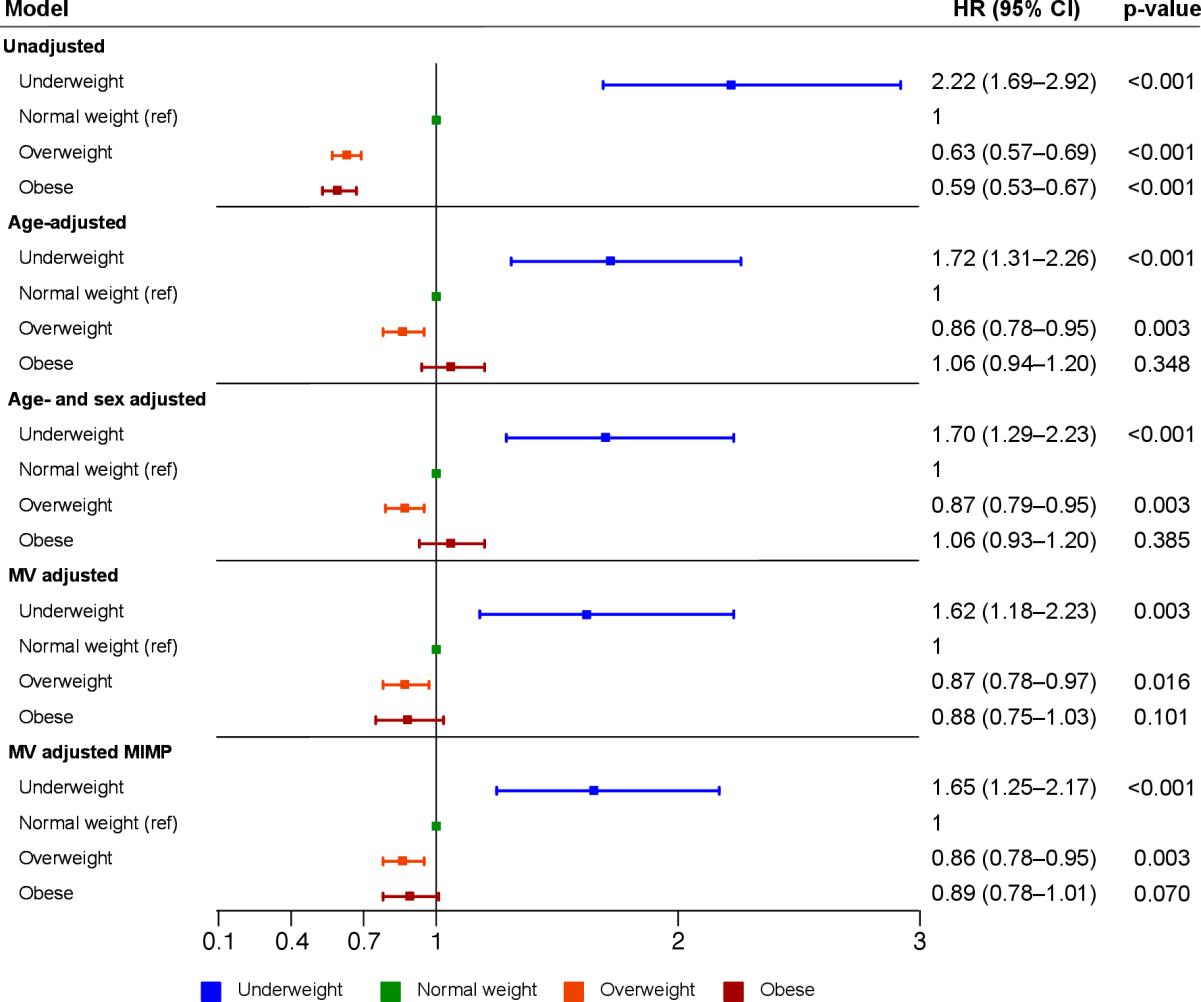
Unadjusted and adjusted risk for mortality (95% CI) in patients with STEMI using log-10 scale for the x-axis.

Figure 3 presents a Forest plot of HRs displayed in log-10 scale for *1-year* all-cause mortality in different BMI categories using the normal weight population as a reference group. People with underweight had the highest mortality compared with people of normal weight in both unadjusted and multi-adjusted analysis. In contrast, patients with overweight had a lower mortality risk compared with normal weight in both unadjusted and multi-adjusted analyses. Patients with obesity had a lower risk for death than normal weight subjects in unadjusted analysis, but this difference became non-significant in the multi-adjusted analysis considering age, sex and other covariates. Still the HR did not differ much from the overweight group and it is possible that the lack of significance is a type II statistical error.

**Figure 4:**
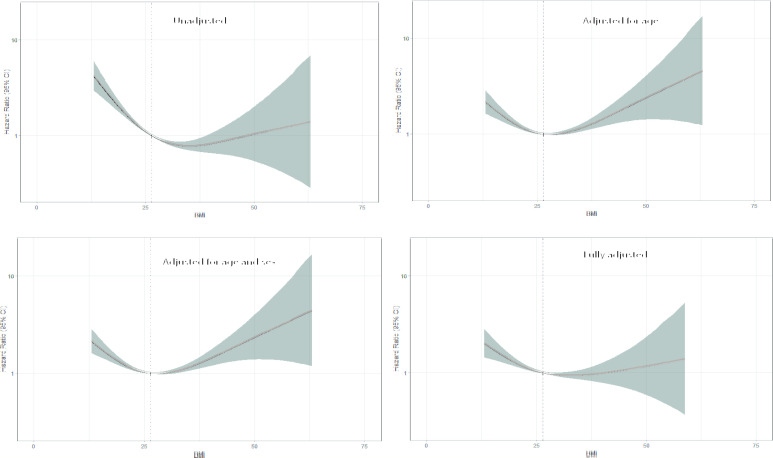
Unadjusted and adjusted fractional polynomial Cox proportional-hazards regression (95% CI, shaded area) with continuous risk relationship between body mass index and all-cause mortality after percutaneous coronary intervention treatment for STEMI.

In figure 4, we examined the association between BMI as a continuous variable and unadjusted and adjusted all-cause mortality using fractional polynomial Cox regression. In models adjusted for age and sex, the curves were U-shaped with a BMI/risk nadir between 25.0 and 29.9 kg/m^2^. In the unadjusted and fully adjusted model, the right side of the curve flattened with wide CIs. In an interaction analysis, the relationship between BMI categories and risk of death was similar in subgroups of selected baseline characteristics (table 2).

**Table 2:** HRs for the risk of mortality in subgroups

Subgroups	BMI category	HR (95% CI)	P-value for interaction
Sex			
Male	Underweight	2.074 (1.272 to 3.383)	0.68
	Overweight	0.873 (0.756 to 1.008)	
	Obese	0.890 (0.730 to 1.084)	
Female	Underweight	1.394 (0.917 to 2.119)	
	Overweight	0.866 (0.721 to 1.039)	
	Obese	0.864 (0.691 to 1.082)	
Age			
>65	Underweight	1.595 (1.140 to 2.230)	0.38
	Overweight	0.896 (0.794 to 1.011)	
	Obese	0.911 (0.771 to 1.076)	
≤65	Underweight	1.964 (0.714 to 5.406)	
	Overweight	0.687 (0.495 to 0.952)	
	Obese	0.709 (0.492 to 1.023)	
Diabetes mellitus			
Diabetic	Underweight	2.591 (1.051 to 6.385)	0.62
	Overweight	0.937 (0.736 to 1.194)	
	Obese	0.877 (0.674 to 1.143)	
Non-diabetic	Underweight	1.535 (1.092 to 2.157)	
	Overweight	0.851 (0.748 to 0.968)	
	Obese	0.898 (0.745 to 1.082)	
Smoking			
Current smoker	Underweight	1.766 (1.017 to 3.066)	0.67
	Overweight	0.782 (0.611 to 1.001)	
	Obese	0.917 (0.674 to 1.249)	
Non-smoker	Underweight	1.550 (1.049 to 2.291)	
	Overweight	0.893 (0.786 to 1.015)	
	Obese	0.871 (0.733 to 1.034)	

There were no significant interactions between dichotomous subgroups and BMI categories with respect to 1-year mortality.

BMI, body mass index.

## Discussion

In the present study, unadjusted statistical analysis showed that people with obesity (BMI≥30.0 kg/m^2^) had the lowest 30-day and 1-year all-cause mortality after coronary intervention due to STEMI, when compared with the subjects of normal weight (BMI 18.5–24.9 kg/m^2^). Thus, in these analyses, our results were in line with the obesity paradox. However, after adjustment for age and sex, the effect of obesity on mortality did not differ from that observed in people of normal weight. Instead, when age and sex had been considered, people with overweight (BMI 25–29.9 kg/m^2^) had the lowest mortality. In the 1-year model adjusted for all covariates, people with overweight still had the lowest death rate, whereas the risk in people with obesity did not differ from that of those with normal weight. However, increasing BMI as a continuous variable in the fully adjusted 1-year analysis, the risk curve flattened out with a wide CI, making it difficult to interpret. Of notice, people with obesity do have a lower mortality in unadjusted analysis and did not display a higher mortality than those of normal weight in the fully adjusted model. On the other hand, people who were underweight (BMI<18.5 kg/m^2^) had a mortality that was substantially higher than that observed for other BMI classes.

Previous publications, including a report from the Framingham heart study,^19^ results from the Canadian APPROACH register^20^ and data from the SCAAR register^12^ showed that in patients with established coronary artery disease, the lowest adjusted risk for mortality reached a nadir around a BMI of 35 kg/m^2^. Furthermore, a meta-analysis by Wang *et al*^21^ found lower all-cause mortality after myocardial infarction in a pooled group of overweight and obese subjects compared with people of normal weight. In contrast, Shahim and coworkers^22^ found no relationship between BMI and myocardial infarction size, or 1-year rates of death or heart failure hospitalisation, in a meta-analysis based on 2238 patients undergoing PCI.

The present study differed from the publications cited earlier regarding several aspects. Our patient population was considerably more homogenous compared with that in previous studies. The heterogenicity of previous study populations involved inclusion of patients undergoing PCI for STEMI, also subjects with NSTEMI treated with PCI or medication alone and in some cases management with coronary artery bypass grafting. Furthermore, in some of these studies, patients with underweight were excluded and adjustment for covariables was not performed as they were not available. It is not unlikely that these differences may explain the main disparity between previous reports and the present study, which showed that the lowest risk for 1-year all-cause mortality after adjusting for covariates was observed among overweight patients (BMI range 25.0–29.9 kg/m^2^). Our findings are more in line with those of Flegal *et al*^23^ who performed a meta-analysis including 2.9 million people and observed that the overweight group had a trend for better survival as compared with those who were obese.

In the present study, underweight individuals (BMI <18.5 kg/m^2^) displayed the highest risk for all-cause 30-day and 1-year mortality post-PCI in both unadjusted and adjusted statistical analyses. The separation between the mortality curves for the underweight group, as compared with other BMI categories, occurred early after PCI and showed, thereafter, a steeper upward slope during 1-year follow-up. We speculate that underweight patients may have an underlying pathophysiology that may generate a larger STEMI, more complications or impaired recoverability than subjects with a higher BMI. Also, previous epidemiological studies have observed a U-shaped relationship between BMI and all-cause mortality. 24 25 This has mainly been attributed to smoking,^26^ respiratory illnesses^27^ and other underlying diseases.^28^ In the present study, underweight patients were more often smokers and had a higher prevalence of COPD. Thus, excessive mortality in underweight patients following PCI for STEMI could also be related to a higher occurrence of underlying comorbidities.

The relationship between categories of BMI and outcome was consistent across all subgroups studied for selected baseline characteristics. Hence, there was no difference between the two sexes, those with age below or above 65 years, those with or without diabetes mellitus, or those who smoked and those who did not.

In a review from 2017, Lavie *et al*^29^ have listed several possible reasons or biases for the obesity paradox including younger patients, fewer smokers, better energy reserves, increased muscle mass and reverse causality due to frailty and cachexia in patients who are leaner, apart from other and unknown confounders. Of notice is that BMI as a measure of obesity does not differ between fat, muscle mass and skeletal weight.^30^ It is highly probable that an augmented muscle mass may act as a protective factor with respect to outcome after coronary interventions.^31^ Lavie *et al* studied patients with stable coronary artery disease and found that those with a higher lean body mass had better survival irrespective of their degree of fatness.^32^ Thus, the importance of muscle mass as an explanatory mechanism of the obesity paradox has probably been underestimated. Also, cardiorespiratory fitness is an important variable that may greatly influence the relationship between obesity and survival after PCI.^33^

### Strengths and limitations

The strength of our study includes a large sample size of real-world data and a homogenous group of patients treated with PCI for STEMI. The main limitation is the observational nature of the study, which precludes us from making any causal inferences. As only surviving hospitalised patients are included, the possibility of selection bias, residual confounding and survival bias cannot be ruled out. BMI, as a measure of obesity, has its limitation as a measure of obesity since it does not distinguish between fat, muscle mass and skeletal weight. Neither waist circumference nor other measures of abdominal fatness were available in the SCAAR and, therefore, we were not able to take into account the distribution of body fat in our analyses. The role of unintentional weight loss was not controlled for and cause-specific mortality data were not studied. Data on death were collected by crosslinking the SCAAR with the Swedish Cause of Death Register, which is a high-quality virtually complete register of all deaths in Sweden since 1952, but is not adjudicated to establish cardiac versus non-cardiac causes of death.

## Conclusions

Although people with obesity displayed lower mortality after treatment with PCI for STEMI as compared with a reference group with normal weight, the two groups showed similar outcomes after relevant covariates were considered. Assessed against the referent group, overweight patients showed the lowest 30-day and 1-year adjusted mortality risk, and underweight individuals the highest. We speculate that the amount of muscle mass and cardiorespiratory fitness may affect the relationship between BMI and outcome in patients with coronary artery disease.

## Data Availability

Data are available on reasonable request.
